# Consequences of neglected traumatic spinal cord injuries

**DOI:** 10.1016/j.jtumed.2022.09.017

**Published:** 2022-10-10

**Authors:** Faisal M. Konbaz, Sami I. AlEissa, Abdulrahman Y. AlHabeeb, Thamer S. AlHowaish, Ghada S. Alhamed, Emad M. Masuadi, Majed S. Abalkhail, Fahad H. AlHelal

**Affiliations:** a Department of Orthopedic Surgery, King Faisal Specialist Hospital & Research Centre, Riyadh, KSA; b Department of Orthopedic Surgery, Ministry of the National Guard – Health Affairs, Riyadh, KSA; c King Abdullah International Medical Research Center, Riyadh, KSA; d King Saud Bin Abdulaziz University for Health Sciences, Riyadh, KSA; e Department of Medical Education, Collage of Medicine, King Saud Bin Abdulaziz University for Health Science, Riyadh, KSA

**Keywords:** الآثار, الكسر, اهمال, النتائج, الإصابة, العمود الفقري, TSCI, Traumatic spinal cord injury, MVA, Motor vehicle accidents, SCI, Spinal cord injury, Consequences, Fracture, Neglect, Outcomes, Spine, Traumatic

## Abstract

**Objectives:**

Spinal cord injuries cause major disabilities and are devastating events for both patients and healthcare providers. Most traumatic spinal cord injuries (TSCIs) are due to motor vehicle accidents (MVAs). Neglected injuries result in complications and poor outcomes. Here, we investigated the causes, consequences, and outcomes of neglected TSCIs.

**Methods:**

This case series study was performed at King Abdulaziz Medical City, Riyadh, KSA. Of the 750 patients treated between February 2016 and February 2021, 18 patients met our inclusion criterion of neglected high-energy TSCI with neurological deficit, necessitating surgical intervention more than 14 days after the index trauma.

**Results:**

Of the 18 patients with neglected TSCIs, 72.2% were men. The patients’ mean age at the time of injury was 36.8 years, 77.8% were from outside Riyadh, and all patients had MVA-induced TSCIs, 88.9% of which were attributable to delayed referral to a tertiary center. The mean duration of neglect was 43 days, and the longest duration was 125 days. The most common site of injury was the thoracolumbar region (55.5%). The American Spinal Injury Association impairment scale score improved in two patients. Bed sores occurred in 55.5%, and deep vein thrombosis occurred in 27.8% of patients. Postoperatively, 77% of patients required intensive care unit admission. Most patients (12) did not receive specialized spinal cord injury rehabilitation postoperatively.

**Conclusion:**

Early referral of patients with TSCIs is crucial to prevent short- and long-term complications.

## الملخص

**أهداف البحث:**

تسببت إصابات الحبل الشوكي بإعاقات كبيرة تعتبر أحداثا مدمرة ومحبطة لكل من المرضى ومقدمي الرعاية الصحية. معظم إصابات الحبل الشوكي الرضحية تنسب الى الحوادث المرورية. الإصابات المهملة تؤدي الى مضاعفات ونتائج سيئة. في هذا الدراسة نبحث في أسباب وعواقب ونتائج إصابات الحبل الشوكي الرضحية المهملة

**طرق البحث:**

تم إجراء هذه الدراسة في مدينة الملك عبد العزيز الطبية في الرياض، المملكة العربية السعودية. من بين ٧٥٠ مريضا تم علاجهم في الفترة بين فبراير ٢٠۱٦ الى فبراير ٢٠٢۱ استوفى ۱٨ مريضا معيار التضمين الخاص بالدراسة وهي إصابة رضحية عالية الطاقة للحبل الشوكي مصاحبة لعجز عصبي، مما استلزم تخل جراحي بعد۱٤ يوم أو أكثر من تاريخ الإصابة.

**النتائج:**

من بين ۱٨ مريضا يعانون من إصابات الحبل الشوكي الرضحية المهملة، كان ٧٢٫٢٪ منهم ذكور. كان متوسط عمر المرضى وقت الإصابة ۳٦٫٨ عاما، كان جميع المرضى يعانون من إصابات في النخاع الشوكي ناتجة عن حوادث مرورية، وكان ٧٧٫٨٪ من خارج مدينة الرياض والتي تعزى الى تأخر الإحالة الى مركز ثلاثي متخصص عند معظم الحالات (٨٨٫٩٪). بلغ متوسط مدة الإهمال ٤۳ يوما، واطولها ۱٢٥ يوما. كان أكثر مواقع الإصابة شيوعا هو منطقة الصدر القطني (٥٥٫٥٪). تحسن لدى ٢ من المرضى مقياس الضعف بناء على مقياس جمعية إصابات العمود الفقري الأمريكية. حدثت تقرحات الفراش لدى ٥٥٫٥٪ وتجلط الأوردة العميقة في ٢٧٫٨٪ من المرضى. بعد الجراحة، احتاج ٧٧٪ من المرضى الى دخول العناية المركزة. لم يتلق معظم المرضى (۱٢) تأهيل متخصص لإصابات النخاع الشوكي بعد الجراحة

**الاستنتاجات:**

الإحالة المبكرة للمرضى الذين يعانون من إصابات الحبل الشوكي الرضحية ضرورية لمنع المضاعفات القصيرة والطويلة -الأجل.

## Introduction

Spinal cord injury (SCI) refers to any trauma that results in permanent or temporary alterations in the normal sensory, motor, and autonomic functions of the spinal cord.^1^ SCIs are considered catastrophic events for both patients and healthcare providers.^2^ Traumatic spinal cord injuries (TSCIs) are attributable to several etiological factors including motor vehicle accidents (MVAs), falls from height, gunshot injuries, sport-induced traumas, and falls from a load overhead.^3^ MVAs account for most cases of TSCI globally^4^ and have been implicated as the leading cause of TSCI in KSA.^5^ The estimated annual global incidence of TSCI is 40–80 cases per million people, according to World Health Organization statistics.^6^ TSCI leads to loss of sensory and motor function below the level of the injury, thus often resulting in spasticity, autonomic dysreflexia, and many other SCI-induced phenomena.^7^ Early post-injury intervention is associated with better functional outcomes,^1^ and surgical options are limited in patients who present late.^8^

Neglected TSCIs are defined as injuries that are discovered late and do not receive timely multidisciplinary treatment because limited therapeutic options are feasible as a result of the delay. In Western countries, neglected or delayed treatment of TSCIs are usually attributable to missed injuries at the time of initial presentation. In addition to missed injuries, delayed presentation to the appropriate service is an important contributor to delayed or neglected TSCIs in developing countries. In this case series, the terms “neglected” and “delayed” TSCIs are used interchangeably to describe TSCIs in patients who underwent surgical treatment at our hospital >14 days after the index trauma.^3^ Missed injury refers to injuries in patients with concomitant head injury or multiple traumas that might have led to delayed SCI diagnosis (overlooked diagnosis).^9^ The exact definition of neglected SCI remains under debate. Hassan et al. have defined a neglected SCI as an injury in which the interval between the cervical SCI and accurate diagnosis exceeds 3 weeks.^8^

## Materials and Methods

**Figure 1:**
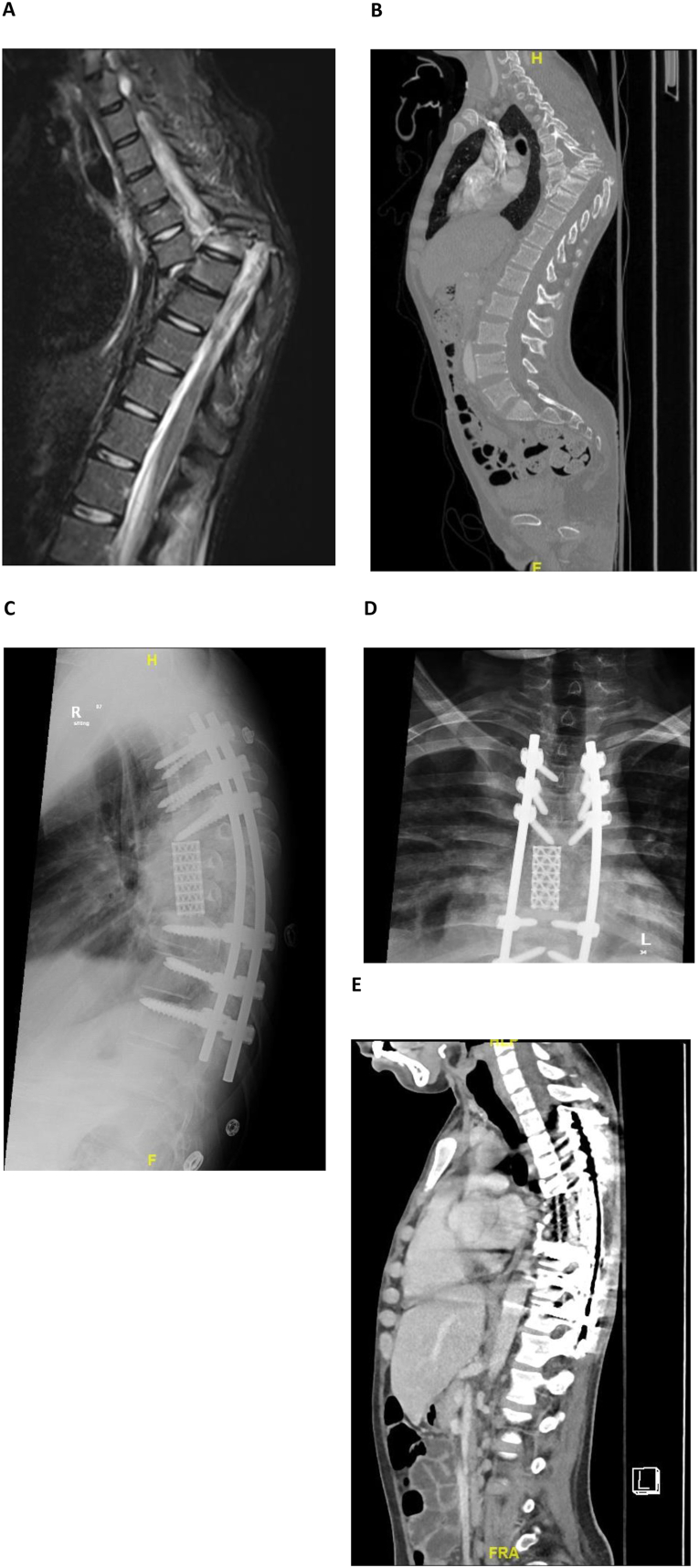
Young patient with neglected T6/7 fracture dislocation with severe kyphotic deformity and overlap (A, B) MRI and CT images showing the severity of the spinal cord injury and spinal column. (C, D, E) X-ray and CT postoperative images showing reconstruction surgery with three levels of corpectomy, multiple levels of fixation with pedicle screws, and reconstruction with a mesh cage.

This case series study was performed at King Abdulaziz Medical City, a tertiary center in Riyadh, KSA, after IRB approval was granted by the King Abdullah International Medical Research Center. Of the 750 patients who visited the hospital between February 2016 and February 2021, 18 patients met our inclusion criterion of any neglected high-energy TSCI concomitant with neurological deficit. Neglect was defined as a management delay ≥14 days after the injury. Patients <18 years of age were excluded from the study. Data were obtained and reviewed manually by using the BESTCare system, an integrated electronic medical record system that provides patient data, including demographics, injuries, management, neglect, complications, hospital course, and consequences. Furthermore, we evaluated neurological status by using American Spinal Injury Association (ASIA) scores pre- and postoperatively.^1^ We recorded 42 independent variables in each patient. Access to data was limited to the research team, and we ensured that patient privacy and confidentiality were not violated. Information regarding identifiers was not obtained, and all data (both printed and electronic) were stored in a secure system within the Ministry of the National Guard Health Affairs and King Abdulaziz Medical City premises. All statistical analyses were performed in SPSS software (see Fig. 1).

## Results

Of the 18 patients with neglected TSCI, 72.2% were men. The patients’ ages at the time of injury ranged from 18 to 77 years (mean age 36.8 years). The mean body mass index was 28.8 kg/m^2^. Notably, 77.8% of the patients were from outside Riyadh, the capital of KSA. The mechanism of injury was MVA in all patients. Causes of neglect were categorized into the following groups: (a) delay in referral to a tertiary hospital, (88.9% of cases), (b) initial patient refusal to undergo surgical intervention (5.6% of cases), and (c) missed diagnosis at first contact (5.6% of cases) (Table 1). The mean duration of neglect was 43 days (range 14–125 days) (Table 2).

**Table 1:** Causes of neglect.

Cause of neglect	N	Percentage
Delay in referral to tertiary center	16	88.9
Patient refusal of surgical intervention	1	5.6
Missed diagnoses	1	5.6

**Table 2:** Durations.

	N	Minimum	Maximum	Mean	Std. deviation
Duration of neglect in days	18	14	125	43.0	31.3
Days from injury to hospital discharge	18	42	264	126.7	58.4
Length of stay from admission to discharge	18	23	250	97.0	55.6
Days from injury to start of rehabilitation	6	33	110	64.2	27.0

We identified eight cervical injuries and ten thoracolumbar injuries. Most cervical injuries were treated with the anterior approach (five patients); the posterior approach was used in one patient; and a combined anterior and posterior approach was used in two patients. The posterior approach was used in ten patients with thoracolumbar injuries: six patients underwent pedicle screw fixation with or without laminectomy, and the remaining four patients underwent more complex surgical fixation with posterior pedicle screw fixation and corpectomy through a posterior approach. Notably, four patients underwent multiple spine surgeries during the same admission for various reasons: two patients required irrigation and debridement to treat surgical site infection, and two patients underwent a planned second-stage procedure to complete spine fracture fixation. Most (11) patients had multiple injuries in addition to spine fractures. The mean estimated blood loss in patients with cervical injuries was 261.1 mL, and that in patients with thoracolumbar injuries was 561.1 mL. The mean operation time was 343.8 min.

The ASIA score remained unchanged in 15 patients. However, the scores improved from grades B to C, or from C to D in two patients (Table 3). The ASIA score was unavailable in one patient who was intubated after a severe head injury. Bed sores developed in 55.5% of the patients, and six patients developed bed sores before they presented to our hospital. Deep vein thrombosis (DVT)/pulmonary embolism (PE) occurred in 27.8% of patients before presentation to our hospital. We observed infection during hospitalization in six patients, and one patient who was transferred from a previous hospital presented with infection at the time of admission; most patients had urinary tract infections (UTIs), and two patients developed surgical site infections necessitating surgical debridement and intravenous antibiotic administration. Postoperatively, 14 (77%) patients were admitted to the intensive care unit (mean intensive care unit stay 17.6 days). No patients commenced a SCI rehabilitation program before surgical intervention, and only six patients underwent specialized SCI inpatient rehabilitation postoperatively after a mean delay of 64.2 days. Unfortunately, most patients (12) did not undergo specialized SCI inpatient rehabilitation after surgical intervention but instead continued to attend an outpatient rehabilitation program.

**Table 3:** ASIA scores at admission and discharge.

			ASIA score at discharge^1^				Total
			A	B	C	D	
ASIA score at admission^1^	A	Count	11	0	0	0	12
		% of total	64.7%	0.0%	0.0%	0.0%	70.6%
	B	Count	0	1	1	0	1
		% of total	0.0%	5.9%	5.9%	0.0%	5.9%
	C	Count	0	0	0	1	1
		% of total	0.0%	0.0%	0.0%	5.9%	5.9%
	D	Count	0	0	0	3	3
		% of total	0.0%	0.0%	0.0%	17.6%	17.6%
Total		Count	11	1	1	4	17
		% of total	64.7%	5.9%	5.9%	23.5%	100.0%

Only two patients (11.1%) returned to their work/education; seven patients (38.8%) were unable to return to work because of the injury. Furthermore, three patients did not work before the injury, and data from six patients were unavailable.

## Discussion

TSCI, a devastating event for both healthcare providers and patients, poses major physical, emotional, and financial burdens,^10^ and occurs after an MVA in most cases.^11^, ^12^, ^13^ The incidence of SCI in KSA is among the highest globally (62.37 cases per million).^14^ This high incidence is attributable to the high incidence of MVAs in KSA. The mortality risk is higher in people who experience TSCI than in other populations; unfortunately, MVA-induced mortality rates are as high as 254 per million in KSA, as compared with 95 per million in Australia and 152 per million in the United States.^15^^,^^16^ Typically, TSCI casualties are men in their 30s or 40s.^17^

Early rehabilitation is the primary goal of TSCI management. Several studies have shown that early rehabilitation decreases the length of hospitalization and facilitates earlier restoration of functional independence.^18^, ^19^, ^20^ In a retrospective study of 123 patients, Sumida et al.^18^, ^19^, ^20^, ^21^ have observed that early rehabilitation improves the ASIA motor score (a measure of functional independence) and decreases the length of hospitalization. Another retrospective study has categorized patients into three groups on the basis of the interval between the injury and admission for acute rehabilitation: a short interval (<30 days), moderate interval (31–60 days), and long interval (60 days) between injury and admission. The authors observed a significant change in daily living outcomes in the group with a short interval until admission, in association with greater independence.^22^

Despite the delayed presentation, all 18 patients in our study underwent surgical intervention to achieve spinal stability before initiation of rehabilitation. Optimal stability is aimed at better pain control, improved nursing care, prevention of post-traumatic spinal deformities, and preservation of any residual neurological function.

Two international studies have investigated neglected SCI. An Indian study including 61 patients with neglected SCIs has reported that neglected SCIs were most common in individuals between 20 and 35 years of age, and were predominantly observed in men. The most common cause of injury was falls from height, followed by MVAs. The duration of neglect was >24 weeks in most patients, and the lower cervical spine was the most common site involved.^3^ In another retrospective study involving 40 patients with neglected thoracolumbar TSCIs, inadequate treatment at the first contact hospital was the most common cause of neglect. The most common complication was pressure sores, which were extremely severe in some cases and resulted in osteomyelitis of the underlying bone.^9^

We also confirmed patients’ return-to-work status and observed that most patients in our study did not return to work/education, thus potentially indicating the extent of disability and its possible effects on independence.

Similarly to the findings of previous studies, in this study, the highest prevalence of neglected TSCIs was observed in men.^3^^,^^9^^,^^14^^,^^16^^,^^17^ Most patients resided outside Riyadh, the capital city. Chhabra et al. have reported similar findings, in which 55.74% of the patients in the study lived in rural areas. Those authors have reported that falls from height were the predominant mechanism of injury. In contrast, MVA-induced injury was observed in all patients in our study.^3^ The most common cause of delay in our study was delayed referral to a tertiary hospital. In contrast, other studies have reported premature discharge during the first admission or inadequate management as predominant causative factors in developing countries, and missed diagnosis as the predominant cause in Western countries.^3^^,^^9^^,^^23^^,^^24^ The mean duration of neglect was 43 days in our study, whereas most patients included in the study by Chhabra et al. presented after 168 days. Thoracolumbar injury was the predominant injury in our study, whereas cervical spine injury was predominant in the study by Chhabra et al.^3^ The isolated posterior approach for thoracolumbar injuries was used in 60.0% of the patients in our study, similarly to findings by Khatri et al., who have used this therapeutic approach in 65.0% of patients with thoracolumbar injuries.^9^ We observed expected neurological improvement in two patients in our study. However, Khatri et al. have reported 52.5% improvement by at least 1 point in the ASIA score, although the scores in the other categories remained unchanged.^9^ Bed sores occurred in 55.5% of patients in our study, as compared with 62.3% of patients in the study by Chhabra et al. and 58.0% of patients in the study by Khatri et al. Notably, half the bedsores observed in patients occurred before presentation to our hospital, thus highlighting the importance of early surgical intervention and subsequent rehabilitation to prevent such complications. Similarly, DVT and PE occurred in five patients who were diagnosed with these life-threatening complications of neglected TSCI at the time of presentation to our hospital; the DVT incidence was 27.8% in our study, a finding similar to those in previous studies investigating neglected TSCIs. We observed several cases of UTI, a common complication associated with all types of TSCI regardless of the time until management.^25^ No patients in our study commenced the SCI rehabilitation program before surgical intervention; this finding is similar to those observed in most patients by Chhabra et al.^3^ Only one-third of the patients in our study underwent an inpatient rehabilitation program, whereas the remaining patients underwent less extensive rehabilitation on an outpatient basis. Further large-scale studies are warranted to investigate whether delayed presentation might contribute to bypassing the inpatient rehabilitation phase in patients with TSCIs. Table 4 shows a comparison between our study and those by Chhabra et al. and Khatri et al.

**Table 4:** Comparison of this study, Chhabra et al.,^3^ and Khatri et al.^9^

		This study	Chhabra et al.	Khatri et al.
Number of cases		18	61	40
Mechanism of injury	MVA	100%	39.34%	NA
	Fall from height	0%	47.54%	NA
Cause of neglect	Delayed referral	88.9%	NA	NA
	Premature discharge at first admission	NA	52.5%	45%
	Missed diagnosis	5.6%	4.9%	17%
Duration of neglect		Mean duration 43 days	Majority presented at > 168 days	
Improvement in ASIA score^1^		11.7%	NA	52.5%
Bed sores		55.5%	62.3%	58%
DVT		27.8	8.2%	26%
UTI		27.7%	50.82%	42%
Initiation of rehabilitation	Yes	33%	6.6%	NA
	No	66%	93.4%	NA

Our results indicated that most patients were from peripheral or rural areas, thus explaining some of the factors responsible for the treatment delays in these patients. Beyond delays in referral, as expected, people rural areas may lack awareness regarding the importance of early management of traumatic spinal cord injuries and may fear spine surgeries. Moreover, in some cases, a lack of advanced equipment or personnel such as spine or trauma surgeons might contribute to the delay. A shortage of specialized physicians can also delay referral to specialized hospitals, because of missed radiological diagnosis or simply improper routine neurological examination of patients. Additionally, improper management of accidents and emergency units at hospitals in rural areas may contribute to delays. Proper guidelines are essential to manage patients during referral to specialized hospitals. Furthermore, false notions that, because neurological recovery is unlikely, early surgical intervention is not necessary or important might also cause the delay in management or referral. Many solutions are available to address this issue, including employing more specialized trauma surgeons in rural hospitals, providing advanced equipment necessary to better manage patients, and increasing awareness regarding early management of traumatic spinal cord injuries and how spine surgeries can prevent further deterioration.

In this study, we investigated the consequences of delayed treatment of TSCIs in patients in the Middle East. We observed that delayed referral was the primary contributor to delays in management, and we identified various potential complications associated with delayed treatment of TSCIs. Furthermore, we highlighted the challenges associated with rehabilitation of these patients. We recommend that similar studies be performed across KSA and the Middle East to determine areas requiring improvement to provide optimal treatment of patients with TSCIs, to emphasize the importance of establishing a system that enables timely transfer of patients with TSCIs and hiring qualified personnel. This study has several limitations, specifically the small sample size, short duration of follow-up, and retrospective study design. These factors decreased the accuracy of analysis of the causes underlying delays in transfer of patients to the appropriate hospital setting. Moreover, we were unable to accurately analyze the effects of concomitant injuries in patients with TSCIs, and we did not compare neglected TSCIs with TSCIs treated in a timely manner.

## Conclusions

Early referral of patients with TSCIs is essential to ensure prompt rehabilitation to prevent short- and long-term complications.

## Funding source

This research did not receive any specific grant from funding agencies in the public, commercial, or not for-profit sectors.

## Conflict of interest

The authors have no conflict of interest to declare.

## Ethical approval

This research was approved by King Abdullah International Medical Research Center (KAIMRC) (study number: NRC21R.014.01).

## Author contributions

FMK was responsible for choosing the topic, conducting the literature search, screening potentially eligible studies, reviewing the data, interpreting results, writing the manuscript, and reviewing the manuscript.

SIE was responsible for writing and reviewing the manuscript.

AYH was responsible for writing the protocol and report, conducting the literature search, screening potentially eligible studies, extracting and analyzing data, updating reference lists, writing the manuscript, and reviewing the manuscript.

TSA was responsible for writing the protocol and report, conducting the literature search, screening potentially eligible studies, extracting and analyzing data, updating reference lists, writing the manuscript, and reviewing the manuscript.

GSH was responsible for designing the review protocol, conducting the literature search, screening potentially eligible studies, extracting and analyzing data, updating reference lists, writing the manuscript, and reviewing the manuscript.

EMM was responsible for cleaning and analyzing the data.

MSA was responsible for interpreting the results.

FHH was responsible for writing and reviewing the manuscript.

All authors have critically reviewed and approved the final draft and are responsible for the content and similarity index of the manuscript.
